# Psychometric properties of the Spanish version of the functionality appreciation scale

**DOI:** 10.1186/s40337-024-01004-0

**Published:** 2024-04-25

**Authors:** Ángel Zamora, Lorena Desdentado, Rocío Herrero, Marta Miragall, Rosa Baños

**Affiliations:** 1 Polibienestar Research Institute, University of Valencia, Calle Serpis 29, 46022 Valencia, Spain; 2 Department of Personality, Evaluation, and Psychological Treatments, University of Valencia, Av. Blasco Ibañez 21, 46010 Valencia, Valencia Spain; 3 CIBER of Physiopathology of Obesity and Nutrition (CIBEROBN), Instituto de Salud Carlos III, Av. Monforte de Lemos, 3-5, 28029 Madrid, Spain; 4 Department of Psychology and Sociology, University of Zaragoza, Teruel, Spain

**Keywords:** Functionality appreciation, Positive body image, Test adaptation, Factor structure, Invariance, Spanish

## Abstract

**Background:**

The Functionality Appreciation Scale is a 7-item measure of an individual’s appreciation of his or her body for what it can do and is capable of doing. While this instrument has been increasingly used in intervention-based research, its psychometric properties have not been extensively studied in non-English-speaking populations. The psychometric properties of a novel Spanish translation of the FAS were examined.

**Methods:**

An online sample of 838 Spanish adults (mean age = 31.79 ± 11.95 years, 50.48% men) completed the Spanish FAS and validated measures of body appreciation, eating disorder symptomatology, intuitive eating, and life satisfaction.

**Results:**

Exploratory factor analysis supported a 1-dimensional factor structure of the FAS, which was further supported by confirmatory factor analysis (SBχ²(14) = 83.82, SBχ²_normed_ = 1.48, robust RMSEA = 0.094 (90% CI = 0.074, 0.115), SRMR = 0.040, robust CFI = 0.946, robust TLI = 0.924). Invariance across genders was shown, and there were no significant differences according to gender (*t*_(417)_ = 0.77, *p* =.444, *d* = 0.07). Construct validity was also supported through significant associations with the other measures of the study. Incremental validity was established in women. Thus, appreciation of functionality predicted life satisfaction over and above the variance accounted for by other body image and eating disorder-related measures (*F*_(4, 399)_ = 18.86, *p* <.001, Δ*R*^2^ = 0.03).

**Conclusions:**

These results support the psychometric properties of the Spanish FAS and demonstrate the importance of the appreciation of functionality in relation to a healthier body image and psychological wellbeing.

**Supplementary Information:**

The online version contains supplementary material available at 10.1186/s40337-024-01004-0.

## Abstract (plain language summary)

Research has shown that individuals with eating disorders often have a negative body image. To promote a more positive body image, it is recommended to focus on appreciating the functions of the body (functionality appreciation). Functionality appreciation refers to valuing the body for what it can do. The Functionality Appreciation Scale (FAS) is a commonly used tool to assess this variable. However, the scale has not yet been adapted and tested for use in the Spanish population. The study revealed that the Spanish FAS can effectively measure how individuals value their bodies for their capabilities, regardless of gender. Appreciating what your body can do is linked to a better body image, healthier eating habits, and overall well-being. The adapted version of the FAS enables Spanish health professionals to gain a better understanding of their patients suffering from an eating disorder.

**Supplementary Information:**

The online version contains supplementary material available at 10.1186/s40337-024-01004-0.

## Background

Body image (BI) has been defined as thoughts, feelings, perceptions, and behaviors about one’s body [1]. It is a multidimensional construct that includes both negative and positive BI [2]. Traditionally, research on BI has largely focused on the negative or pathological aspects of BI (e.g., body dissatisfaction and body shame) [3]. Focusing on reducing the symptoms of negative BI without enhancing positive aspects may result in a neutral BI at best, which is reflected in statements such as “I don’t hate my body, I just tolerate it” [4]. However, the study of positive BI is still in its infancy, although it is growing exponentially [4]. 

Positive BI can be broadly defined as the love and acceptance of one’s body and is accomplished by appreciating one’s singularity and functionality [4–6]. It is also a multidimensional construct that includes not only satisfaction with body appearance but also other dimensions, such as body functionality, body appreciation or BI flexibility [4].

Specifically, the construct of body functionality (BF) has gained increased amounts of attention in recent years. BF encompasses functions related to physical capacities, internal processes, bodily senses and perceptions, creative endeavors, communication with others, and self-care [7]. Another related but slightly different concept to BF is functionality appreciation, which goes beyond body functionality by including aspects such as appreciation, respect, and honor toward the body for what it is capable of doing [8]. The functionality appreciation is considered a key dimension in achieving a more complete and holistic understanding of BI [9].

According to a recent meta-analysis [10] functionality appreciation has been shown to be negatively associated with body mass index (BMI), body dissatisfaction, body surveillance, eating pathology, and general distress. Otherwise, it has been shown to be positively associated with body acceptance by others, body esteem, BI flexibility, body satisfaction, interoceptive awareness, self-compassion, self-esteem, and well-being-related constructs such as life satisfaction. Moreover, there were no significant differences according to gender. In addition, functional appreciation is a predictor of both the continued absence of seven core eating disorder symptoms, such as purging, fear of weight gain, or overeating [11], and greater intuitive eating [12].

In the assessment of positive BI, a novel study using item pool visualization has shown that while the Body Appreciation Scale (BAS-2) [13, 14] may be the most accurate measure of overall positive BI, a combination of this measure with a body pride or functionality appreciation measure is needed for broader coverage of this construct [15]. Similarly, the most widely used instrument to measure functionality appreciation is the Functionality Appreciation Scale (FAS) [8], which has been shown to capture facets of positive BI that differ from body appreciation, body acceptance from others, BI flexibility, positive rational acceptance, and body pride [15].

The FAS is a 7-item questionnaire that originally showed a unidimensional solution [8]. During the last few years, the unifactorial structure of the FAS has been replicated in adults [16–27], adolescents [20, 28], and LGBQ populations [29, 30], revealing divergent, convergent, criterion, and incremental validity, as well as adequate test-retest reliability and internal consistency. Moreover, the stability of psychometric properties across genders has been reported in adult samples from Japan [23], China [20], Malaysia [24, 31], Iran [28], Lebanon [25], Romania [26], Italy [18], the Republic of Cyprus [17], the Netherlands [16], Colombia [22], the United States of America [8], and the United Kingdom [31].

Despite the amount of evidence demonstrating the adequate psychometric properties of the FAS in samples from different countries, to the best of our knowledge, there is no adapted or validated version of the FAS in the Spanish population. Hence, the present study aimed to adapt and test the psychometric properties of the FAS by examining factor structure, evidence of validity, and reliability in an adult Spanish sample. Additionally, invariance across genders was tested. We hypothesized that the Spanish version of the FAS would show a 1-factor structure in both exploratory and confirmatory factor analyses, which would be invariant across genders, enabling the examination of gender differences in FAS scores. Based on the findings mentioned above, we did not expect to find significant differences in the FAS score between genders. In terms of convergent validity, we hypothesized that the FAS would show significant positive associations with measures of body appreciation, intuitive eating, and life satisfaction but significant negative associations with eating disorder symptoms and BMI. Finally, we expected that the FAS would demonstrate incremental validity by significantly predicting life satisfaction beyond the effects of body appreciation, eating disorder symptoms, and intuitive eating.

## Methods

### Participants

A sample of 838 Spanish individuals from the general population was recruited online (415 women and 423 men, age range: 18–71; BMI range 13.33–44.44). The demographic information for the sample is described in Table 1.

**Table 1:** Descriptive statistics of the sample (*N* = 838)

Characteristic	Total sample (*n* = 838) n (%)	Women (*n* = 415) n (%)	Men (*n* = 423) n (%)
**Age**	31.79 (11.95)	31.5 (11.77)	32.08 (12.14)
**BMI**	23.97 (4.12)	23.2 (4.21)	24.72 (3.89)
**Marital status**			
Single	406 (48.44%)	208 (50.12%)	198 (46.81%)
Married/Civil partner/Couple	409 (48.81%)	191 (46.02%)	218 (51.54%)
Divorced/Separated	18 (2.15%)	12 (2.89%)	6 (1.42%)
Widower	5 (0.6%)	4 (0.96%)	1 (0.2%)
**Educational level**			
Primary education	13 (1.55%)	6 (1.45%)	7 (1.65%)
Secondary education	155 (18.51%)	65 (15.66%)	90 (21.28%)
University studies (higher education/university degree)	421 (50.24%)	208 (50.12%)	213 (50.35%)
University studies (master’s degree)	215 (25.66%)	113 (27.23%)	102 (24.11%)
University studies (doctorate)	34 (4.06%)	23 (5.54%)	11 (2.60%)
**Occupation**			
Student	278 (33.18%)	146 (35.18%)	132 (31.21%)
Active worker	468 (55.87%)	222 (53.49%)	246 (58.16%)
Unemployed	59 (7.05%)	28 (6.75%)	31 (7.33%)
Sick leave	7 (0.83%)	3 (0.72%)	4 (0.95%)
Retired or early retiree	10 (1.19%)	4 (0.96%)	6 (1.42%)
Other	16 (1.91%)	12 (2.89%)	4 (0.95%)

Note. *SD* = standard deviation; BMI = body mass index

### Procedures

Ethics approval for this study was obtained from the ethics committee of Arnau Vilanova Hospital (5.3 CEI, 30_2021). All the data were collected via a Qualtrics survey between February and June 2023. The inclusion criterion for participation was being an adult resident and citizen of Spain. The study was published on social media and included an estimated duration. After providing informed consent, the participants were asked to complete the instruments described above, including attentional control questions, to ensure the quality of the responses (e.g., if you are reading this carefully, mark “sometimes”). The assessment automatically ended if the questions were not adequately answered. Participants completed the survey voluntarily and received feedback on their responses (i.e., their total scores on the questionnaires) and psychoeducational material (i.e., an explanation of the constructs assessed with the questionnaires) via email. In addition, an email address was provided for participants to contact the researchers with questions or doubts about the study or the feedback on their scores. Specifically, 171 males were recruited through a paid platform and redeemed with 5€ for completing the survey to balance the sample in terms of gender.

### Measures

#### Demographics

Participants reported their gender identity, age, educational level, occupation, marital status, height, and weight. Height and weight data were used to compute the BMI as kg/m2.

#### Functionality appreciation

The functionality appreciation was measured with a Spanish translation of the FAS [8]. All items were rated on a 5-point scale ranging from 1 (strongly disagree) to 5 (strongly agree). The FAS was translated into Spanish using a five-stage procedure [32], which has been recommended for adapting BI questionnaires [33]. This procedure involved [1] the independent forward translation of the FAS by two bilinguals [2], the production of a synthesized forward translation [3], the back translation by two new bilinguals working independently, and [4] committee evaluation of the forward and back translations with the original author of the questionnaire. The only minor concern related to item 7 was that there may be a nuanced difference between ‘performs’ (original item) and ‘fulfills’ (back-translation). The former conveys a sense of merely performing an action, whereas ‘fulfills’ implies adherence to a specific standard or objective. Considering this, a prefinal version was tested for understanding (1 = *I do not understand at all*, 5 = *I understand completely*) in a community sample of 28 Spanish individuals (53.6% women; age: *M* = 31.32, *SD* = 10.9, range = 18–64). The overall mean scores of these items (*M* = 4.55, *SD* = 0.47, range = 3.57-5) suggested a high degree of understanding of the translated items. Therefore, the committee approved the final version of the Spanish FAS without any further revisions. The FAS items in English and Spanish are reported in Table 2.

#### *Body appreciation*: body appreciation Scale-2 (BAS-2) [13, 14]

The BAS-2 is a 10-item instrument that assesses acceptance of one’s body, respect and care for one’s body, and protection of one’s body from unrealistic beauty standards. All the items were rated on a 5-point scale ranging from 1 (*never*) to 5 (*always*). The overall score was computed as the mean of the scores for all the items. Higher scores on this scale reflect greater body appreciation. The Spanish version of the BAS-2 has shown adequate psychometric properties [14]. In the present study, McDonald’s ω for the BAS-2 scores was 0.95 (95% CI = 0.94, 0.95).

#### *Eating disorders symptomatology*: eating attitudes scale (EAT-26) [34]

The EAT-26 was used to measure the symptoms and concerns characteristic of eating disorders. The EAT-26 comprises the following three dimensions: (1) dieting (13 items relating to distortion of BI), (2) bulimia (6 items regarding BI and tendency toward bulimic behavior), and (3) oral control (7 items referring to self-control and high-risk behaviors associated with anorexia nervosa). All the items were rated on a 6-point Likert scale (1 = never; 6 = always). The total score is calculated as the sum of the item scores, ranging from 0 to 78, with higher scores reflecting more severe eating disorder symptomatology. The Spanish version of the EAT-26 has shown adequate psychometric properties and a one-factor structure [35]. In the present study, McDonald’s ω for the EAT-26 scores was 0.88 (95% CI = 0.89, 0.93).

#### *Intuitive eating*: intuitive eating scale (IES-2) [36]

The IES-2 includes 23 items assessing the four domains of intuitive eating: (1) unconditional permission to eat (UPE; 6 items); (2) eating for physical reasons rather than for emotional reasons (EPR; 8 items); (3) reliance on hunger and satiety cues (RHSC; 6 items); and (4) body-food choice congruence (B-FCC; 3 items). Each item is rated on a 5-point Likert scale (1 = strongly disagree; 5 = strongly agree). The Spanish version of the scale showed adequate psychometric properties [37], although a higher-order factor structure has not yet been studied in this population. In the present study, McDonald’s ω for the IES-2 subscales were as follows: UPE: 0.76 (95% CI = 0.76, 0.82); EPR: 0.88 (95% CI = 0.88, 0.93); RHSC: 0.89 (95% CI = 0.87, 0.90); B-FCC: 0.81 (95% CI = 0.78, 0.88).

#### *Life satisfaction*: satisfaction with life scale (SWLS) [38]

The SWLS comprises five items rated on a 7-point Likert scale (1 = Strongly disagree; 7 = Strongly Agree). The total score was calculated by adding all item scores, with higher scores indicating greater life satisfaction. The Spanish version has shown adequate psychometric properties [39]. In the present study, McDonald’s ω for SWLS scores was 0.89 (95% CI = 0.87, 0.91).

### Analytic strategy

#### Data treatment

There were no missing values in the dataset, as the online survey required responses to all items to complete the survey. Significant differences between genders in terms of sociodemographic variables were tested. The results indicated a significant difference in BMI, with men having a higher mean *(t*_(836)_ = 5.45; *p* <.001; *d* = 0.37). However, there were no significant differences for age, marital status, occupation, and educational level. In addition, we analyzed significant differences in all assessed variables between financially compensated and non-compensated male participants (see Supplementary 1).

The factor structure of the Spanish FAS was examined using exploratory factor analysis (EFA) to confirmatory factor analysis (CFA) [33]. To ensure adequate sample sizes for both EFA and CFA, we split the main sample using a random seed, resulting in a split-half for the EFA (women *n* = 208, men *n* = 211) and another split-half for the CFA (women *n* = 207, men *n* = 212). There were no significant differences between the two split-half subsamples for age, *t*_(836)_ = 1.18, *p* =.239, *d* = 0.08; for BMI, *t*_(833)_ = 0.94, *p* =.346, *d* = 0.06; or for the men/women ratio, χ^2^_(1)_ = 1.28, *p* =.261.

#### Exploratory factor analysis

To explore the factor structure of the FAS items, we computed two EFAs with the first split-half subsample using the *psych* package [40] in R 4.2.2 [41]. Two EFAs were run separately for women and men following the methodology of Alleva et al. [8]. Our sample size satisfied the Worthington & Whittaker [42] item-communality requirements (i.e., ≥ 0.50), as well as assumptions for EFA based on item distributions, average item correlations, and item-total correlations [43]. Data factorability was assessed using the Kaiser–Meyer–Olkin (KMO) measure of sampling adequacy (which should ideally be ≥ 0.80) [44] and Bartlett’s test of sphericity (which should be statistically significant). The EFAs were estimated using the principal axis, as the results are similar to those of maximum likelihood estimation without assuming multivariate normality [45], which cannot be assumed in our samples (Kolgomorov-Srminov test for all items: *p* <.001; Mardia’s skewness = 718.76, *p* <.001; Mardia’s kurtosis = 26.17, *p* <.001). The number of factors to be extracted was based on the results of the parallel analysis. Following the methodology of Alleva et al. [8], a varimax rotation was applied. The items were retained in accordance with Comrey & Lee’s recommendation (loadings ≥ 0.33) [46]. In addition, the degree of factor similarity across genders was assessed using Tucker’s congruence coefficient [47], with values between 0.85 and 0.94 corresponding to fair similarity across groups and values ≥ 0.95 suggesting that factor structures can be considered equal across groups [48]. Moreover, we followed the recommendation of Swami et al. [33] and Montoya & Edwards [49] to also examine the results of the parallel analysis by retaining only those factors with an eigenvalue greater than the eigenvalue from the random data [50].

#### Confirmatory factor analysis

We used the second split-half subsample to conduct a CFA using the *Lavaan* [51], *semTools* [52], and *MVN* packages [53] with R [41]. Previous Monte Carlo simulations with different seed values and based on factor loadings reported by Alleva et al. [8] have indicated that a sample size of approximately 180 is sufficient for this analysis [18], which was surpassed in this subsample. Based on previous studies examining the factor structure of the FAS, we hypothesized that our EFA would suggest a 1-factor structure model, which would be subsequently tested in the CFA. Since univariate and multivariate normality could not be assumed (Kolgomorov-Smirnov test for all items: *p* <.001; Mardia’s skewness = 702.33, *p* <.001; Mardia’s kurtosis = 23.31, *p* <.001), the model was computed using the robust maximum likelihood method, and fit indices with the Satorra–Bentler correction were applied [54]. Specifically, we used the normed model chi-squared (χ²/df; values < 3.0 considered indicative of good fit), the Steiger–Lind root mean square error of approximation (RMSEA) and its 90% CI (values equal to or less than 0.06 considered indicative of good fit and up to 0.08 indicative of adequate fit), the standardized root mean square residual (SRMR; values < 0.08 indicative of good fit), the Tucker–Lewis index (TLI; values close to or > 0.95 indicative of good fit), and the comparative fit index (CFI; values close to or > 0.95 indicative of adequate fit) [55]. Additionally, evidence of convergent validity was assessed in this subsample using the Fornell–Larcker criterion [56], with average variance extracted (AVE) values ≥ 0.50 considered adequate [57], indicating that a latent variable is able to explain more than half of the variance of its indicators on average (i.e., items converge into a uniform construct).

#### Gender invariance

To test the measurement invariance of the FAS across genders in the Spanish population, that is, the equivalence of its factorial structure between Spanish women and men, we computed a multigroup CFA [58]. Measurement invariance was assessed at the configural, metric, scalar, and strict levels [59]. Configural invariance implies that both genders have the same indicators (items) for the latent variable(s) (i.e., the unconstrained model should fit the data well in both groups). In addition to configural invariance, metric invariance implies that factor loadings are equivalent across genders. In addition to configural and metric invariance, scalar invariance implies that item intercepts are similar across genders. Finally, strict invariance implies, in addition to all previous invariance levels, that the residual variances are similar across genders [58].

Invariance across groups was established when the results of the chi-square tests between models were not significant. In addition, when this criterion was marginally met or not met, we consulted changes in model fit indices according to Chen’s criteria (ΔCFI ≥ − 0.010 and ΔRMSEA ≥ 0.015 or ΔSRMR ≥ 0.030) for further insight and a more convincing and practical assessment of noninvariance.

Finally, we aimed to test for gender differences in the FAS scores using an independent-samples *t*-test only if scalar or partial scalar invariance was established.

#### Reliability and validity

Internal consistency in both genders was estimated using McDonald’s ω and its associated 95% CI, with values greater than 0.70 reflecting adequate internal reliability [60]. McDonald’s ω was selected as a measure of composite reliability because of known problems with the use of Cronbach’s α [61]. The hierarchical ω was computed using the *semTools* package for *R* [52], which allows for models that do not fit the data perfectly [62].

The construct validity of the Spanish FAS scores was examined through bivariate correlations between scores on the FAS and BAS-2, IES, EAT-26 and SWLS. Correlations between FAS scores and age and BMI were also examined, as in previous research. These analyses were conducted separately for women and men and interpreted according to Cohen’s criterion [63], with Pearson correlation coefficients of ∼ 0.10 considered a weak correlation, ∼ 0.30 considered a moderate correlation, and ∼ 0.50 considered a strong correlation.

Incremental validity was assessed by examining whether FAS scores predicted SWLS scores beyond the variance accounted for by body appreciation, symptoms of disordered eating, and intuitive eating. For this purpose, hierarchical linear regression models were computed and were supported if we found a statistically significant increase in the adjusted *R*^2^ when the FAS score was included as a predictor.

## Results

### Exploratory factor analyses

#### Factor analysis with women

For women, Bartlett’s test of sphericity, χ^2^(21) = 759.35, *p* <.001, and KMO (0.91) indicated that the FAS items had adequate common variance for factor analysis.

The EFA results revealed a single factor with λ > 1 (λ1 = 3.94, λ2 = 0.19), and parallel analysis revealed that one factor from the actual data had a greater λ than the criterion λ generated from the simulation (λ_1_ = 3.94 > 0.47), which explained 56% of the common variance.

All 7 items loaded strongly onto the extracted factor, with coefficients ranging between 0.66 and 0.80 (Table 2). The descriptive statistics for each item are presented in supplementary 2.

**Table 2:** Items of the FAS in English and Spanish and factor loadings derived from EFA and CFA

	EFA			CFA	
Item	EFA subsample (*n* = 419)	Women (*n* = 211)	Men (*n* = 208)	CFA subsample (*n* = 419)	
	*M (SD)*	Factor		*M (SD)*	Total
(1) I appreciate my body for what it is capable of doing / *Aprecio mi cuerpo por lo que es capaz de hacer.*	3.87(0.91)	0.74	0.54	3.91(0.91)	0.73
(2) I am grateful for the health of my body, even if it isn’t always as healthy as I would like it to be / *Estoy agradecido/a por la salud de mi cuerpo, aunque no siempre esté tan sano como me gustaría.*	4.07(0.91)	0.71	0.69	4.04(0.88)	0.59
(3) I appreciate that my body allows me to communicate and interact with others / *Aprecio el hecho de que mi cuerpo me permita comunicarme e interactuar con otros.*	4.23(0.85)	0.74	0.78	4.25(0.79)	0.72
(4) I acknowledge and appreciate when my body feels good and/or relaxed / *Reconozco y aprecio cuando mi cuerpo se siente bien y/o relajado/a*.	4.31(0.86)	0.66	0.70	4.33(0.75)	0.65
(5) I am grateful that my body enables me to engage in activities that I enjoy or find important / *Estoy agradecido/a de que mi cuerpo me permita implicarme en actividades que disfruto o que me parecen importantes.*	4.28(0.88)	0.80	0.74	4.35(0.77)	0.76
(6) I feel that my body does so much for me / *Siento que mi cuerpo hace mucho por mí*.	3.93(0.95)	0.78	0.63	3.92(0.96)	0.77
(7) I respect my body for the functions it performs / *Respeto mi cuerpo por las funciones que cumple.*	4.07(0.93)	0.80	0.70	4.15(0.82)	0.78

*Note. EFA* = exploratory factor analysis; CFA = confirmatory factor analysis *M =* mean; *SD* = standard deviation

#### Factor analysis with men

For men, Bartlett’s test of sphericity, *χ*^*2*^(21) = 571.16, *p* <.001, and the KMO test (0.87) indicated that the FAS items had adequate common variance for factor analysis. Principal axis EFA indicated that only one factor had an eigenvalue greater than 1 (λ = 3.31, λ2 = 0.26), and parallel analysis showed that one factor from the actual data had a greater λ than that from the random data (λ_1_ = 3.31 > 0.49), which explained 47% of the common variance.

All 7 items loaded strongly onto the extracted factor, with coefficients ranging between 0.54 and 0.78 (Table 2). The descriptive statistics for each item are presented in Table S1.

#### Factor structure congruence and composite reliability

The factor loadings reported in Table 1 for women and men separately suggest strong similarity across factor structures. Indeed, Tucker’s congruence coefficient (0.99) indicated that factor structure equivalence across the models for women and men can be assumed. McDonald’s ω was adequate for women (0.90, 95% CI = 0.89, 0.92) and men (0.86, 95% CI = 0.83, 0.91), as was the total EFA subsample (0.88, 95% CI = 0.84, 0.91).

### Confirmatory factor analysis

CFA indicated that the fit of the 1-factor model of FAS scores was acceptable: SBχ²(14) = 83.82, SBχ²_*normed*_ = 1.48, robust RMSEA = 0.094 (90% CI = 0.074, 0.115), SRMR = 0.040, robust CFI = 0.946, robust TLI = 0.924. In particular, the RMSEA value and its 90% confidence interval are slightly above the recommended threshold of 0.06, indicating a reasonable model fit.

The standardized estimates of factor loadings ranged from 0.59 to 0.78 (Table 2). The convergent validity of this model was acceptable, as the AVE was 0.51.

#### Composite reliability

The composite reliability of the scores was adequate for women (ω = 0.89, 95% CI = 0.77, 0.91), men (ω = 0.88, 95% CI = 0.76, 0.94), and the total CFA subsample (ω = 0.88, 95% CI = 0.79, 0.89).

### Gender invariance

We tested for measurement invariance across genders for the full CFA subsample. As reported in Table 3, the indices indicated that configural, metric, and scalar invariance were supported, as the results of the chi-square tests were not significant in all the cases.

In particular, the chi-squared test for the strict model was marginally nonsignificant (Δχ² = 13.9, *df* = 7, *p* =.053); therefore, we verified Chen’s (2007) criteria (i.e., ΔCFI ≥ − 0.010 and ΔRMSEA ≥ 0.015 or ΔSRMR ≥ 0.010) and concluded that the Spanish FAS scores showed scalar invariance (ΔCFI = − 0.009, ΔRMSEA = 0.001, ΔSRMR = 0.010).

Therefore, it can be assumed that both genders have the same indicators (items) for the latent variable, with equivalent factor loadings and intercepts. However, the residual variances differ between genders.

In addition, we conducted an independent sample *t*-test to determine gender differences in the FAS scores. The results showed that there were no significant differences in the observed FAS scores between women (*M* = 4.11, *SD* = 0.63) and men (*M* = 4.16, *SD* = 0.65) in the CFA split-half subsample (*t*_(417)_ = 0.77, *p* =.444, *d* = 0.075).

**Table 3:** Measurement invariance across genders in the CFA split-half subsample

Model	SBχ²		Robust CFI	Robust RMSEA	SRMR	Model Comparison	ΔSBχ²	ΔRobust CFI	ΔRobust RMSEA	ΔSRMR	Δdf	
		df										*p*
Configural	71.98	28	0.957	0.097	0.037							
Metric	77.15	34	0.958	0.087	0.047	Configural vs. metric	4.70	− 0.010	− 0.010	0.010	6	0.582
Scalar	86.74	40	0.956	0.082	0.051	Metric vs. scalar	8.67	− 0.002	− 0.005	0.004	6	0.193
Strict	100.20	47	0.947	0.083	0.061	Scalar vs. Strict	13.90	− 0.009	0.001	010	7	0.053

Note. SB = Satorra-Bentler; df = degrees of freedom; CFI = comparative fit index; RMSEA = Steiger–Lind root mean square error of approximation; SRMR = standardized root mean square residual

### Evidence of convergent validity and criterion-related validity

To assess the evidence of convergent validity of the FAS, we examined bivariate correlations between the scores on the FAS and the other measures included in the study separately for women and men using the full sample (i.e., the EFA and the CFA split subsamples). For both men and women, appreciation of body functionality was strongly, significantly, and positively correlated with body appreciation. Additionally, moderate to strong positive correlations were found between FAS and all IES-2 subscales in women. The same was observed for men, except for the absence of a significant correlation between FAS and UPE. In addition, the association between functionality appreciation and eating disorder symptomatology was significant only for women (negative and moderate). These results support the convergent validity of the Spanish FAS.

**Table 4:** Bivariate correlations between variables for men (top diagonal) and women (bottom diagonal)

Variable	1	2	3	4	5	6	7	8	9	10
(1) FAS		0.60**	0.25**	0.09	0.23**	0.40**	− 0.03	0.42**	0.10*	− 0.19**
(2) BAS	0.70**		0.40**	0.15**	0.30**	0.43**	− 0.12*	0.59**	0.15**	− 0.28**
(3) EPR	0.32**	0.46**		0.19**	0.21**	0.29**	− 0.20**	0.31**	0.15**	− 0.22**
(4) UPE	0.23**	0.37**	0.24**		0.24**	− 0.04	− 0.34**	0.08	− 0.10*	− 0.13*
(5) RHSC	0.34**	0.40**	0.34**	0.34**		0.24**	− 0.01	0.16**	− 0.02	− 0.21**
(6) B-FCC	0.44**	0.38**	0.28**	0.02	0.34**		0.06	0.37**	− 0.08	− 0.22**
(7) EAT-26	− 0.33**	− 0.49**	− 0.44**	− 0.48**	− 0.27**	− 0.14**		− 0.04	− 0.12*	− 0.01
(8) SWLS	0.50**	0.53**	0.27**	0.22**	0.27**	0.20**	− 0.33**		0.09	− 0.17**
(9) Age	0.04	0.11*	0.19**	− 0.16**	− 0.03	− 0.03	− 0.22**	0.13**		0.30**
(10) BMI	− 0.21**	− 0.26**	− 0.23**	− 0.21**	− 0.14**	− 0.18**	0.14**	− 0.04	0.22**	

*Note. FAS = Functionality Appreciation Scale, BAS = Body Appreciation Scale; EPR = Eating for Physical rather than Emotional Reasons; UPE = Unconditional Permission to Eat; RHSC = Reliance on Hunger and Satiety Cues; B-FCC = Body-Food Choice Congruence; EAT-26 = Eating Attitudes Test; SWLS = Satisfaction With Life Scale; BMI = body mass index;* ^*^ *p* *<.05;* ^**^ *p* *< −.001.*

Regarding the evidence of concurrent validity, the associations between functionality appreciation and life satisfaction were significant, positive, and strong for both genders. Additionally, we found weak significant and negative associations between BMI and functionality appreciation in both genders and a weak and positive association with age in men (see Table 4 for the remaining correlations).

### Evidence of incremental validity

To test for evidence of incremental validity, we conducted separate hierarchical linear regressions for women and men with life satisfaction as the criterion variable and all the other variables as the predictor variables in the first step and functionality appreciation as a predictor in the second step (see Table 5 for full regression coefficients).

For women, the first step of this regression was significant, *F*_(6, 397)_ = 27.30, *p* <.001; Adj. *R*^2^ = 0.28, as was the second step, *F*_(7, 396)_ = 27.37, *p* <.001; Adj. *R*^2^ = 0.31. The addition of functionality appreciation in the second step accounted for a significant incremental change in the Adj. *R*^2^, *F*_(1)_ = 18.86, *p* <.001, Δ*R*^2^ = 0.03; emerging as a significant predictor of life satisfaction.

For men, the first step of the hierarchical regression was significant, *F*_(6, 404)_ = 39.75, *p* <.001, Adj. *R*^2^ = 0.36. The second step of the regression was also significant, *F*_(7, 403)_ = 34.22, *p* <.001, Adj. *R*^2^ = 0.36 (Table 5). However, the addition of functionality appreciation in the second step did not account for a significant incremental change in the amount of variance explained by step 1 (*F*_(1)_ = 1.05, *p* =.306, Δ*R*^2^ = 0).

Thus, evidence of incremental validity was found only in women, as FAS scores were significantly and positively associated with SWLS after excluding the shared variance with the BAS, IES-2 subscales, and EAT-26.

**Table 5:** Results of multiple hierarchical regression analyses for the prediction of life satisfaction

		Women (*n* = 415)						Men (*n* = 423)				
Step	Variable	**B**	SE		β	*t*	*p*	**B**	SE	β	*t*	*p*
1	BAS	.36	.04		.47	8.53	<.001	.44	.04	.52	10.95	<.001
	EPR	.01	.36		0	.02	.986	.56	.34	.07	1.65	.101
	UPE	−.24	.42		−.03	−.56	.573	.21	.42	.02	.49	.626
	RHSC	.56	.36		.08	1.55	.121	−.38	.34	−.05	-1.09	.276
	B-FCC	−.14	.38		−.02	−.37	-71	1.07	.36	.14	3	.003
	EAT-26	−.07	.04		−.09	-1.68	.093	.03	.05	.03	0.65	.518
2	BAS	.23	.05		.31	4.65	<.001	.41	.05	.49	8.79	<.001
	EPR	.06	.35		.01	.17	.863	.57	.34	.07	1.68	.094
	UPE	−.2	.41		−.02	−.5	.62	.19	.42	.02	.45	.652
	RHSC	.48	.35		.07	1.35	.179	−.39	.34	−.05	-1.12	.261
	B-FCC	−.56	.39		−.07	-1.44	.15	1.01	.36	.13	2.77	.006
	EAT-26	−.07	.04		−.09	-1.68	.093	.03	.05	.03	.61	.54
	FAS	2.56	.57	.27		4.47	<.001	.53	.52	.05	1.03	.306

Note. *FAS = Functionality Appreciation Scale, BAS = Body Appreciation Scale; EPR = Eating for Physical rather than Emotional Reasons; UPE = Unconditional Permission to Eat; RHSC = Reliance on Hunger and Satiety Cues; B-FCC = Body-Food Choice Congruence; EAT-26 = Eating Attitudes Test; SWLS = Satisfaction With Life Scale*; B = nonstandardized regression coefficient; SE = Standard Error; β = Standardized regression coefficient; *t = t* value; *p* = *p* value

## Discussion

The FAS has shown good psychometric properties for measuring appreciation of what the body is capable of doing across a wide range of nations [8, 17–27, 64], adolescents [20, 28], and even social identity groups [29, 30]. However, its psychometric properties have not been previously explored in the Spanish population, which was the aim of the present study. Specifically, our results supported the unidimensional model of the FAS, as confirmed through both EFA and CFA. Moreover, the measurement of this model maintained scalar invariance across genders. Furthermore, the FAS consistently showed adequate composite reliability and adequate evidence of convergent, concurrent, and, in the case of women, incremental validity.

As mentioned before, regarding the factor structure of the Spanish version of the FAS, our EFA results supported the extraction of a unidimensional model consisting of seven items for both genders. Similarly, our CFA results supported a unidimensional model of FAS scores, with good fit indices and factor loadings indicating that all seven items loaded strongly on the hypothesized factor. It is worth noting that the RMSEA value found in our CFA results is slightly higher than the established threshold for adequate fit. However, this value falls within the range found in the rest of the previous FAS validations (0.059 to 0.108) [17, 23]. At this point, it should be mentioned that some authors recommend interpreting the SRMR instead of the RMSEA for assessing the model fit including variables with an ordinal response scale, like the FAS [65]. These findings are consistent with previous psychometric studies of the FAS in different nations and age groups [8, 10, 16–27].

In addition, our results also indicated that the unidimensional model of the FAS achieved scalar invariance across genders, allowing for comparisons between groups despite the lack of strict invariance, which is often difficult to establish [66]. On this basis, we examined gender differences in the FAS scores, which did not reach statistical significance. However, the possible ceiling effect may make it difficult to examine mean differences between groups, as suggested in previous works [26]. In general, these findings are consistent with previous literature indicating that the FAS achieves scalar invariance across genders and that there are no significant gender differences in FAS scores [8, 17, 18, 20, 21, 23, 24]. Other studies have shown significantly greater scores for men [22, 28] and women [16, 25].

The evidence of construct validity of the Spanish FAS was also demonstrated in the present study. In terms of convergent validity, our findings revealed a significantly positive and strong association between functionality appreciation and body appreciation similar to that of [18], in contrast to the moderate association found in previous studies [8, 16–18, 22–25]. This finding supports the need noted by Haliwell [67] to clarify whether the constructs that fall under the umbrella of positive BI may overlap, be inaccurate, or replicate existing aspects. However, recent work has shown that the BAS-2 provides the closest and most accurate measure of a core positive BI construct, whereas the FAS taps more distal aspects [15]. Similarly, functionality appreciation was also generally associated with the intuitive eating dimensions, except for men on the UPE subscale, an absence of correlation already reported in recent work for both genders [23]. Furthermore, the negative associations between functionality appreciation and eating disorder symptoms were also consistent with previous research, although this is true only for women [10]. In previous studies, functionality appreciation has been negatively associated with self-objectification only in women [8], which is a key factor in the development of eating disorders, such as anorexia nervosa or bulimia [68]. However, a negative association between the appreciation of functionality and the internalization of the muscular ideal or appearance orientation has been found in men [8], which are constructs that can lead to dysmorphic disorders that are not covered by the EAT-26 questionnaire, such as muscle dysmorphia [69]. In essence, functionality appreciation appears to be a unique construct linked to a more positive BI, a reduced risk of developing eating disorders, and greater psychological wellbeing, as noted by Alleva et al. [8]

In addition, evidence of concurrent validity was demonstrated, as FAS scores were significantly, positively, and strongly associated with life satisfaction in both genders. In contrast, the evidence for incremental validity was mixed. This finding was supported only for women, as the FAS scores were found to significantly predict life satisfaction after controlling for the effects of body appreciation, intuitive eating dimensions, and eating disorder symptoms. However, FAS scores accounted for only a small portion of the incremental variance in life satisfaction, with body appreciation being the stronger predictor in both genders. These results are consistent with previous literature using life satisfaction as the criterion variable [22]. Therefore, since body appreciation is considered a core facet of positive BI [15], it is not surprising that body appreciation is a stronger predictor of life satisfaction and other well-being indicators (e.g., self-esteem) than functionality appreciation [17].

In summary, this work has shown adequate psychometric properties and scalar invariance of the FAS between genders. Therefore, it could be assumed that the FAS measures functionality appreciation in a similar way across genders, allowing for its use in both women and men and the interpretation of gender differences in this construct. However, it remains unexplored if measurement invariance across other social groups could also be assumed (e.g., age groups). Finally, the results suggest that functionality appreciation may play a role in psychological well-being and maladaptive eating behaviors. This highlights the importance of including functionality appreciation in interventions aimed at improving positive BI and/or psychological well-being, particularly in women. Along these lines, a recent meta-analysis has found seven randomized controlled trials where psychological interventions designed to cultivate functionality appreciation have resulted in greater improvements in this construct than in control conditions [10]. ‘Expand Your Horizon’ [7], a structured writing program designed to enhance functionality appreciation, has shown potential in promoting positive BI not only in a general female sample but also in women experiencing rheumatoid arthritis [70]. This work presents the first adapted and validated version of the FAS for the Spanish population, which paved the way for promoting basic and applied research in this novel field.

However, several limitations should be mentioned. First, the online recruitment of the sample limits the generalization of the results to individuals who are not very familiar or skilled with the use of mobile devices or the internet. In addition, 171 men participated under different circumstances than did the rest of the sample because they received financial compensation. Second, although previous literature has shown that FAS scores remain stable for several weeks, we did not assess test-retest reliability [8, 18]. Third, although previous studies have found significant differences in BI dimensions across sexual orientation and gender identity [71], these aspects were not measured in the sample of this study and therefore, gender identities were not identified. Finally, the ceiling effect found in our sample, similar to that found in previous research [26], forces us to be cautious when interpreting some of our results.

## Conclusions

The Spanish version of the FAS was found to be unidimensional and invariant across genders. Furthermore, this version demonstrated adequate psychometric properties among Spanish adults. Our work further revealed positive associations between functionality appreciation and positive BI and between intuitive eating and psychological well-being. Therefore, the current work represents a preliminary step that opens the field for future research in the Spanish context. Future research should continue to examine the role of functionality appreciation in both general and clinical populations, as well as targeting specific social groups, such as sexual and gender minorities or people living in rural areas.

### Electronic supplementary material

Below is the link to the electronic supplementary material.

Supplementary Material 1

## Data Availability

The dataset generated and analyzed during the current study is available in the Open Science Framework repository, https://osf.io/6hdkq/.

## Declarations

### Author’s information

LD was affiliated with the University of Valencia when the study was conducted and is currently affiliated with the Department of Clinical and Health Psychology, Institute of Psychology and Education, Ulm University, Albert-Einstein-Allee 47, 89,069 Ulm, Germany.

## Abbreviations

AVE: Average variance extracted
B =: nonstandardized regression coefficient
β =: Standardized regression coefficient
BAS-2: Body appreciation scale 2
BF: Body functionality
B-FCC =: Body-Food Choice Congruence
BI: Body image
BMI: Body mass index
CI: Confidence interval
CFI: Comparative fit index
CFA: Confirmatory factor analysis
Df: degrees of freedom
EAT-26: Eating attitudes test
EFA: Exploratory factor analysis
EPR: Eating for Physical rather than Emotional Reasons
FAS: Functionality appreciation scale
IES-2: Intuitive eating scale
KMO: Kaiser–Meyer–Olkin
*M*: mean
*N*: sample size
*p*: *p* value
RHSC: Reliance on Hunger and Satiety Cues
RMSEA: Steiger–Lind root mean square error of approximation
SB: Satorra-Bentler
*SD*: standard deviation
*SE*: standard error
SRMR: Standardized root mean square residual
SWLS: Satisfaction With Life Scale
*t = t*: value
TLI: Tucker–Lewis index
UPE: Unconditional Permission to Eat
