# Supplementary material for: Psychometric properties of the Spanish version of the functionality appreciation scale
Source: J Eat Disord. 2024 Apr 25;12:50. doi: 10.1186/s40337-024-01004-0 (PMC11046768; doi:10.1186/s40337-024-01004-0)
Supplement: Supplementary file 1 — Supplementary Material 1 [file 40337_2024_1004_MOESM1_ESM.docx]

# Supplementary material

**Supplementary 1.** Differences between compensated and noncompensated participants in all variables.

The results revealed significant differences in age, marital status, and educational level. First, the mean age of the compensated participants was lower *(t*_(421)_ = -2.25; *p* = .025; *d* = .22). Second, the compensated group consisted mostly of married/civil partner/couple individuals (58.5%), while the noncompensated group was primarily composed of single individuals (51.2%) (χ^2^ _(3)_ = 8.12; *p* = .043). Third, the compensated group consisted mainly of individuals pursuing higher education/university degrees (57.3%), while the other group had a more even distribution between this category (45.6%) and master's degree studies (27.8%) (χ^2^ _(4)_ = 11.24; *p* = .023).

| **Supplementary 2**  Descriptive statistics for FAS items in all subsamples. | | | | | | | | | | | | | |
| --- | --- | --- | --- | --- | --- | --- | --- | --- | --- | --- | --- | --- | --- |
|  | EFA | | | | | | CFA | | | | | | |
|  | Women (*n*= 208) | | | Men (*n*= 211) | | | Women (*n*= 207) | | | Men (*n*= 212) | | | |
| Item | *M (SD)* | Asym. | *K* | *M (SD)* | Asym. | *K* | *M (SD)* | Asym. | *K* | *M (SD)* | Asym. | *K* |  |
| FAS 1 | 3.81(.99) | -.89 | .53 | 3.94(.82) | -.82 | 1.02 | 3.85(.88) | -.26 | -.77 | 3.96(.93) | -.89 | .60 |  |
| FAS 2 | 4.10(.92) | -1.14 | 1.23 | 4.05(.91) | -1.15 | 1.34 | 4.10(.79) | -.95 | 1.35 | 3.98(.96) | -1.07 | .90 |  |
| FAS 3 | 4.27(.82) | -1.07 | .99 | 4.20(.89) | -1.35 | 2.18 | 4.23(.81) | -1.17 | 2.17 | 4.27(.78) | -1.18 | 2.17 |  |
| FAS 4 | 4.23(.88) | -1.03 | .38 | 4.40(.83) | -1.62 | 2.84 | 4.28(.76) | -.87 | .35 | 4.37(.75) | -1.53 | 3.66 |  |
| FAS 5 | 4.26(.87) | -1.42 | 2.35 | 4.30(.89) | -1.57 | 2.76 | 4.33(.74) | -1.12 | 1.13 | 4.37(.79) | -1.50 | 2.89 |  |
| FAS 6 | 3.93(1) | -.74 | -.09 | 3.92(.91) | -.64 | .25 | 3.89(.98) | -.63 | -.14 | 3.95(.95) | -.70 | .08 |  |
| FAS 7 | 3.97(.97) | -.93 | .41 | 4.18(.88) | -1.09 | 1.08 | 4.10(.79) | -.81 | 1.23 | 4.21(.85) | -1.16 | 1.69 |  |
| *Note*. FAS= Functionality Appreciation Scale; *M*= mean, *SD*= standard deviation; *n* = sample size; Asym. = asymmetry; *K*= kurtosis. | | | | | | | | | | | | | |
